# Identification of a novel locus, *BPH38*(t), conferring resistance to brown planthopper (*Nilaparvata lugens* Stal.) using early backcross population in rice (*Oryza sativa* L.)

**DOI:** 10.1007/s10681-019-2506-2

**Published:** 2019-10-10

**Authors:** C. H. Balachiranjeevi, G. D. Prahalada, A. Mahender, Md. Jamaloddin, M. A. L. Sevilla, C. M. Marfori-Nazarea, R. Vinarao, U. Sushanto, S. E. Baehaki, Z. K. Li, J. Ali

**Affiliations:** 1 Rice Breeding Platform, International Rice Research Institute, DAPO Box 7777, Metro Manila, Philippines; 2 Strategic Innovation Platform, International Rice Research Institute, DAPO Box 7777, Metro Manila, Philippines; 3 Indonesian Center for Rice Research, Sukamandi, Indonesia; 4 Chinese Academy of Agricultural Sciences, Beijing, China

**Keywords:** Brown planthopper, QTL mapping, Resistance, MAS, Early backcross population

## Abstract

Rice is the most important staple food crop, and it feeds more than half of the world population. Brown planthopper (BPH) is a major insect pest of rice that causes 20–80% yield loss through direct and indirect damage. The identification and use of BPH resistance genes can efficiently manage BPH. A molecular marker-based genetic analysis of BPH resistance was carried out using 101 BC_1_F_5_ mapping population derived from a cross between a BPH-resistant *indica* variety Khazar and an elite BPH-susceptible line Huang–Huan–Zhan. The genetic analysis indicated the existence of Mendelian segregation for BPH resistance. A total of 702 high-quality polymorphic single nucleotide polymorphism (SNP) markers, genotypic data, and precisely estimated BPH scores were used for molecular mapping, which resulted in the identification of the *BPH38*(t) locus on the long arm of chromosome 1 between SNP markers 693,369 and id 10,112,165 of 496.2 kb in size with LOD of 20.53 and phenotypic variation explained of 35.91%. A total of 71 candidate genes were predicted in the detected locus. Among these candidate genes, *LOC_Os01g37260* was found to belong to the FBXL class of F-box protein possessing the LRR domain, which is reported to be involved in biotic stress resistance. Furthermore, background analysis and phenotypic selection resulted in the identification of introgression lines (ILs) possessing at least 90% recurrent parent genome recovery and showing superior performance for several agro-morphological traits. The BPH resistance locus and ILs identified in the present study will be useful in marker-assisted BPH resistance breeding programs.

**Electronic supplementary material:**

The online version of this article (10.1007/s10681-019-2506-2) contains supplementary material, which is available to authorized users.

## Introduction

Rice is an essential cereal crop that feeds more than 3.5 billion people globally. There is a need to increase rice production by up to 42% by 2025 to feed the ever-increasing population (Seck et al. 2012; Ray et al. 2013). Additionally, about 52% of world rice production is lost annually because of living organisms, of which approximately 21% alone is contributed by the incidence of insect pests (Khush 1979; Sogawa et al. 2003). Among these insect pests, brown planthopper (BPH; *Nilaparvata lugens* Stal.) is one of the major devastating sap-sucking pests, prevalent throughout the rice-growing countries of Asia (Normile 2008). BPH causes significant damage to the rice crop by sucking sap from the xylem and phloem tissues, which ultimately leads to “hopperburn” and, hence, eventually complete yield loss. Besides the direct damage caused by BPH, it causes indirect damage by transmitting viruses (rice grassy stunt virus and ragged stunt virus) that also results in a yield penalty (Sogawa^1982^; Cha et al. 2008; Cabauatan et al. 2009).

Different pest management strategies are available to manage the damage caused by insect pests, including chemical control, improving field practices, and developing and cultivating resistant varieties. However, excessive use of chemicals for BPH management is hazardous to human health, cost-ineffective; and unsafe for the environment and natural biodiversity. Additionally, these chemicals would increase pest outbreak incidences as they can kill the natural predators and their competitors, which feed on BPH (Tanaka et al. 2000). Alternatively, the most durable, precise, and environmentally friendly strategy is to develop a sustainable host-plant resistance management system, which makes it possible to reduce pest incidence, maintain ecological fitness, and keep infestation below economic threshold levels (Brar et al. 2009).

To date, 37 BPH resistance genes have been reported from different resistance sources (Li et al. 2019; Yang et al. 2019; Yuexiong et al. 2019). Notably, the majority of the BPH resistance genes are mapped on six of the 12 chromosomes (2, 3, 4, 6, 11, and 12) and, reportedly, four clusters of BPH resistance loci are located on three chromosomes. Cluster A is located on the long arm of chromosome12 and contains eight loci. Clusters B and D are located on the short and long arm of chromosome 4 containing 13 and four QTL/gene loci, respectively, including the recently identified *BPH34*, *BPH30,* and *BPH36* resistance genes located on chromosome 4 (Li et al. 2019; Wang et al. 2018; Kumar et al. 2018). Cluster C is located on the short arm of chromosome 6, which includes six gene loci (Fujita et al. 2013). Out of 34 BPH resistance genes, 20 genes were fine mapped and only eight were cloned and characterized (*BPH14, BPH17, BPH18, BPH26, BPH29, BPH9, BPH32,* and *BPH6*) (Du et al. 2009; Tamura et al. 2014; Liu et al. 2015; Wang et al. 2015; Ji et al. 2016; Ren et al. 2016; Zhao et al. 2016; Guo et al. 2018). Wang et al. (2018) mapped *BPH30* by using F_2:3_ populations AC1613/9311 and fine mapped by using backcrossed population on the short arm of the chromosome 4. Later, population derived from Kangwengingzhan/RBPH16 and HHZ/RBPH17 (lines RBHH16 and RBPH17 derived from *O*. *rufipogon* accession GX2183) were used to map *BPH36* and *BPH27* on chromosome 4 [*BPH27* already reported by Huang et al. (2013) on Chromosome 4], and *BPH36* were fine mapped by using backcross population (Li et al. 2019). Recently, Yang et al. (2019) mapped a novel QTL *BPH37* by using F_2:3_ population derived from KWQZ/IR64 on chromosome 1, between RM 302 and YM35 markers. It is already reported that IR64 is harboring a major resistant gene *BPH1* on chromosome 12 and minor QTLs associated with BPH resistance, whereas *BPH37* is a second major resistance locus was reported on chromosome 1. Although, unlike blast resistance genes, the cloned BPH resistance genes encode several kinds of proteins such as lectin kinase (*BPH17*), B3 DNA binding protein (*BPH29*), SCR domain (*BPH32*), and exocyst-localized unknown protein (*BPH6*), three popular BPH resistance genes (*BPH26, BPH18,* and *BPH9*) encode a member of coiled-coil–nucleotide-binding site–leucine-rich repeat (CC-NBS-LRR) (Du et al. 2009; Tamura et al. 2014; Liu et al. 2015; Wang et al. 2015; Ji et al. 2016; Ren et al. 2016; Zhao et al. 2016; Guo et al. 2018). This NBS-LRR protein family is involved in the activation of the salicylic acid (SA) dependent and jasmonic acid pathways that mediate sucking inhibition in the phloem sieve element and exhibits a high degree of resistance against different biotypes in India, Korea, and Japan. Hence, these BPH resistance genes encoding NBS-LRR protein family are being used in breeding programs to develop BPH resistant varieties (Suh et al. 2011; Ji et al. 2016; Jena et al. 2017).

Although significant achievements have been made in host-plant resistance to resist the attack of BPH by identifying and introgressing BPH resistance genes, the quick evolution of virulent BPH populations poses a primary concern (Naik et al. 2018). Hence, there is a need to explore more genetic variants from the diverse novel genes for building resistance to defend against the new virulent BPH populations to ultimately, attain durable and broad-spectrum resistance. To address these challenges, our study focused on (1) characterizing the resistance pattern of the parental lines and identifying novel BPH resistance sources (2) detecting the genomic regions conferring BPH resistance, and (3) selecting the introgression lines(ILs) possessing the highest similarity with the recurrent parent and donor genetic segment from a novel breeding strategy.

## Materials and methods

### Plant materials and development of mapping population

HHZ is a high-yielding *indica* variety, originated from Guangdong Province of China and used as the recurrent parent in our study (www.ricedata.cn/variety/varis/600877.htm). The elite cultivar, Khazar originated from Gilan Province, Iran, was used as the donor parent. Taichung Native 1 (TN1) and IR62 were used as susceptible and resistant checks, respectively, for BPH phenotypic screening.

**Fig. 1:**
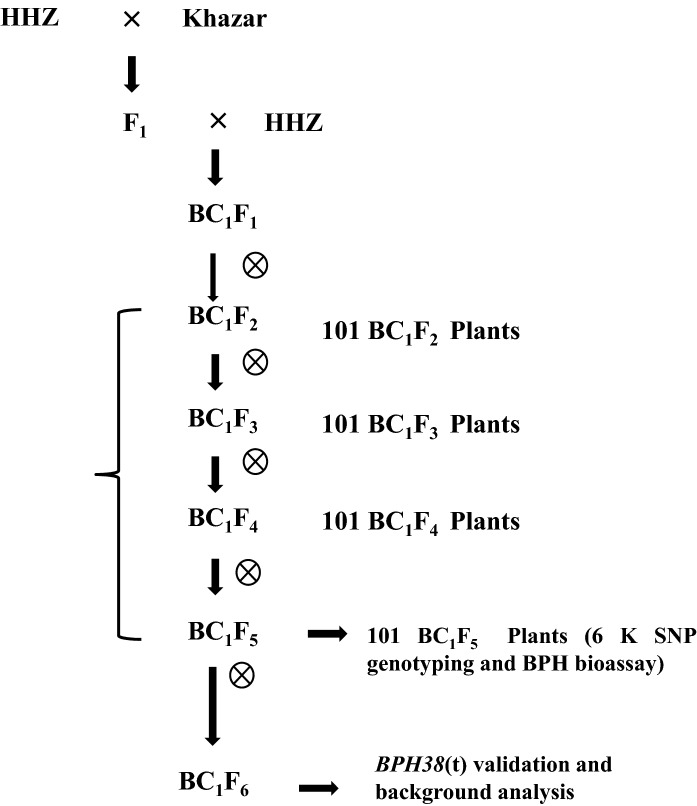
Breeding scheme to develop early backcross mapping population and their utilization

**Fig. 2:**
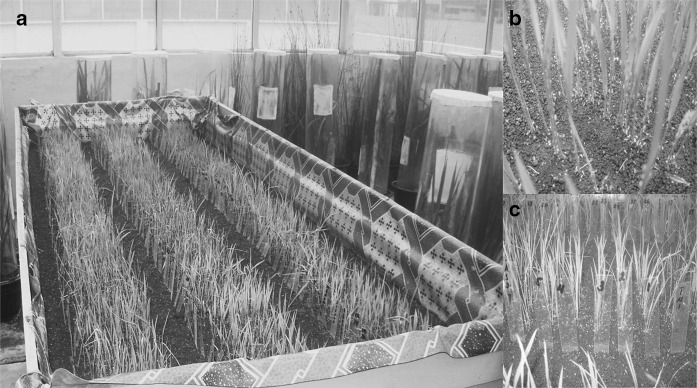
Photographs showing BPH bioassay **a** experimental setup **b** infestation and **c** symptoms, conducted at ICRR, Sukamandi

A cross was made between HHZ and Khazar to generate F_1_ seeds, and the true F_1_ plants were backcrossed with HHZ to obtain BC_1_F_1_ plants. Initially, the derived 101 BC_1_F_1_ plants were subjected to bioassay to study the inheritance pattern of BPH resistance (Table 1). All the 101 BC_1_F_1_ plants were carefully rescued for generating BC_1_F_2_ populations. These BC_1_F_2_ plants advanced by the single seed descent (SSD) method until BC_1_F_5_ mapping population. The 101 BC_1_F_5_ plants generated were subjected to phenotypic and genotypic characterization to explore the hidden genetic variability by identifying the genomic regions conferring BPH resistance. Further, these plants were rescued after bioassay and advanced to BC_1_F_6_ for validation study, background analysis, and phenotypic selection for key agro-morphological traits (Fig. 1). All the 101 BC_1_F_6_ lines were utilized for validation and agronomic traits evaluation. A total of seven single BC_1_F_6_ plants (six plants possessing homozygous dominant allele and one plant possessing homozygous receive allele) were selected out of 101 BC_1_F_6_ lines based on the phenotypic similarity with the recurrent parent and subjected for background genotyping. On the other hand, the same set of 101 BC_1_F_2_ plants and their progenies (BC_1_F_3_) underwent a BPH bioassay at the Indonesian Center for Rice Research (ICRR), Sukamandi, to evaluate the BPH resistance against Sukamandi BPH population (Fig. 2).

**Table 1:** Genetic analysis of BC_1_F_1_ mapping population

S. No	BPH reaction	Number of individuals		O–E	(O–E)^2^	*χ*^2^ = (O–E)^2^/E
		Observed (O)	Expected (E)			
1	R	42	50	8	81	1.62
2	S	59	50	9	81	1.62

### Bioassay for the BPH resistance study

To conduct the BPH bioassay, a local BPH colony collected from Laguna Province and belonging to biotype 3 (Saxena and Barrion 1985) was used. The pure Laguna BPH colony was developed from the single colony of BPH collected from Laguna Province of the Philippines. These colonies were reared and maintained on susceptible cultivar TN1 in the biotic stress screen house (BSSH) facility at IRRI. The bioassay was conducted by following the modified seedbox method (MSB) as described by Jena et al. (2006) at both BC_1_F_1_ and BC_1_F_5_ generations. Seedlings of the mapping population, the parental lines, and resistant and susceptible checks at two- to the three-leaf stage (seven days old) were infested with second- or third-instar nymphs at a density of 10–12 nymphs per seedling. A uniform number of seedlings at two- to three-leaf stage were maintained before the infestation to provide a similar opportunity for all the individuals of the test entries. Once the susceptible check (TN1) started wilting (90%), data on seedling survival rate were recorded and finally expressed as a standard evaluation system (SES) score of 0–9 (IRRI 2014). Higher BPH scores indicate susceptibility and lower scores indicate the resistance response of the genotypes. The scoring pattern for BPH reaction is given in Supplementary Table 1 as mentioned in Prahalada et al. (2017).

### High-resolution genotyping

The Infinium high-density SNP genotyping platform, 6 K SNP chip (Thomson et al. 2017), was used to carry out genotyping of the mapping population. This platform detects SNP alleles by adding fluorescence-labeled, allele-specific nucleotide (allele-specific hybridization) based on single-base extension and detection based on fluorescent color (https://www.ncbi.nlm.nih.gov/pmc/articles/PMC5577349/). Genotyping data were generated in a HapMap format. The obtained polymorphic SNP markers were filtered for no calls and unexpected mono- and heteromorphic SNPs between parents (Prahalada et al. 2017), and prepared according to the QTL IciMapping ver.4.0 input file requirement for linkage map construction and QTL analysis.

### Linkage map construction and QTL mapping

A total of 702 high-quality SNP data points, filtered out from the 6 K SNP chip, were used to construct the linkage map. The Kosambi mapping function was used to convert recombination fraction into mapping distance to anchor markers to the specified location (Kosambi 1944). The linkage map construction was determined by using the QTL analysis software, QTL IciMapping version 4.0 (Meng et al. 2015). Further, QTL mapping was carried out by using the precisely estimated phenotypic BPH SES scores and genotypic data on SNP markers. Composite interval mapping, which is based on a multiple regression model of maximum likelihood with a 1000 permutation test at *P* = 0.05, was used for the QTL analysis as it increases the statistical power and precision of the detected QTLs (Doerge and Churchill 1996; Prahalada et al. 2017; Mahender et al. 2019). LOD of 3.00 was considered as the threshold parameter for determining the significant QTLs among the detected QTLs.

### Post-QTL analysis

In silico post-QTL analysis was performed based on the available information in rice databases: the MSU rice genome annotation project (https://rice.plantbiology.msu.edu/) and RiceXpro (https://ricexpro.dna.affrc.go.jp/). In order to predict the candidate genes associated with a BPH resistance reaction, the candidate genes within the detected locus were listed based on the physical position of the flanking markers. Among the candidate genes, the one that was reported to be involved in biotic stress resistance was considered as a probable candidate gene conferring BPH resistance.

### Evaluation of mapping populations for agro-morphological traits

A total of 101 BC_1_F_6_ plants derived from 101 BC_1_F_5_ mapping population along with its parents were evaluated for the agro-morphological traits: days to 50% flowering (DFF), plant height (PH), tiller number (TN), grain yield (GY), and plot yield (PY). For the evaluation, a randomized complete block design (RCBD) was laid out, and the experiment was carried out with two replications in the 2017 wet season (WS). Each entry was planted in three rows, with 12 plants in each row having a space of 15 × 20 cm between the plants and the rows. DFF was recorded based on the number of days from sowing to 50% flowering in a population on a whole-plot basis. PH was measured from the base of the plant to the tip of the tallest tiller. TN was counted from each plant to express the average TN per plant. The data were recorded from 10 competitive plants from each plot that are chosen at random, and the mean values were computed for different lines. GY per plant and 33 plants per plot were weighed after proper drying (moisture content 12%) as described in Balachiranjeevi et al. (2018).

### Statistical analysis

To analyze the extent of variation of the agro-morphological traits among the test lines, descriptive statistics showing the range, standard deviation (SD), and standard error of the mean (SEm) at *P* = 0.05 were used. The RCBD experimental design was used for conducting the bioassay and the agro-morphological evaluation considering four and two replications, respectively. Analysis of variance (ANOVA) was employed to assess the significance of the bioassay and agro-morphological evaluation at the level of significance *P* = 0.05. DMRT and Fisher’s t-test were used to determine the significant differences of the individual test entries for BPH resistance. Chi-square goodness of fit was used to analyze the segregation pattern of the markers and the phenotypic SES score. The multiple linear regression model of maximum likelihood was used to localize the genomic regions conferring BPH resistance (Prahalada et al. 2017).

## Results

### Bioassay and genetic analysis for BPH resistance

**Fig. 3:**
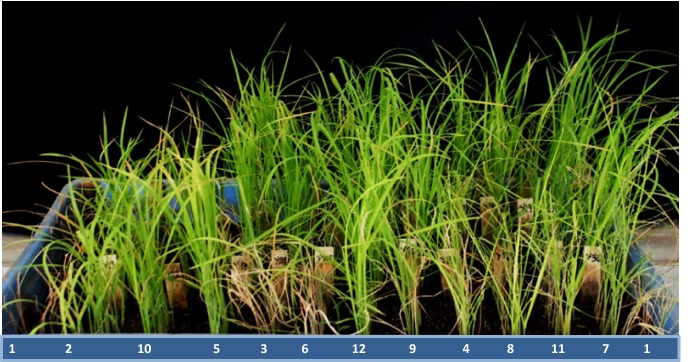
Photograph showing different levels of BPH resistance among the test lines in BPH bioassay on 9th day after BPH infestation. The numbers 1 to 12 shows the BPH reaction of sample test lines (1) susceptible check, TN1; (2) donor parent, Khazar; (3) recurrent parent, HHZ; and (7) resistant check, IR62; (4) to (6) and (8) to (12) are BC_1_F_5_ individuals of mapping population lines

In order to study the mode of segregation of the BPH resistance derived from Khazar, genetic analysis was carried out. The parental lines (HHZ and Khazar) and their 101 derived BC_1_F_5_ lines along with the resistant check IR62 and susceptible check TN1 were evaluated for BPH resistance using the MSB method during the WS of 2017. The parental lines HHZ and Khazar scored 9 and 3, indicating a susceptible and resistance BPH reactions, respectively (Fig. 3). Additionally, chi-square goodness of fit was employed to fit the BPH SES scores of 101 BC_1_F_1_ mapping populations for normal Mendelian segregation. Among 101 BC_1_F_1_ plants, 42 and 59 plants showed resistance and susceptible reaction respectively; and non-significance for the observed and expected ratios, indicating Mendelian inheritance (χ^2^_(0.52)_ = 1.62; Table 1). Hence, from these results, it is evident that a single gene/locus confers BPH resistance derived from Khazar.

### Construction of a high-resolution linkage map

A total of 4606 SNPs extracted from the Infinium 6 K SNP chip were processed before subjecting them to linkage map construction and QTL analysis. SNP markers processing involved filtration for no-call SNPs, unexpected heterozygous, and monomorphic SNPs between parents. A total of 195, 45, and 3664 SNP markers were found to be no calls, unexpected heterozygous and monomorphic SNP markers, respectively. Hence, these markers were filtered out to retrieve a total of 702 high-quality polymorphic SNP markers for further analysis (Table 2). These sets of markers were also analyzed for segregation distortion and were found that all these markers followed Mendelian segregation at the *P* = 0.05 level of significance.

**Table 2:** Filtration of SNP markers extracted from Infinium 6 K SNP genotyping platform

S. No	Description	Total number of initial SNP markers	Total number of SNP markers after filtration
1	Initial markers	4606	–
2	Not amplified markers	195	4411
3	Heterozygous markers	45	4366
4	Monomorphic markers	3664	702
Total polymorphic markers extracted			702

Furthermore, the filtered high-quality polymorphic SNP markers were used to construct the high-resolution linkage map. A total of 702 SNP markers were assigned to different chromosomes depending on the linkage distance. Chromosome 1 was covered by as many as 96 polymorphic markers. The highest number of SNP markers was found on chromosome 1, followed by chromosomes 3, 6, 7, 8, 11, 2, 5, 9, 4, 10 and 12. Apart from identifying the resolution of each chromosome, the percent polymorphism of SNP markers for each chromosome was also studied. The highest percent of polymorphism was attained for chromosome 1, followed by chromosomes 3 and 6 (Supplementary Table 2).

### QTL analysis

**Fig. 4:**
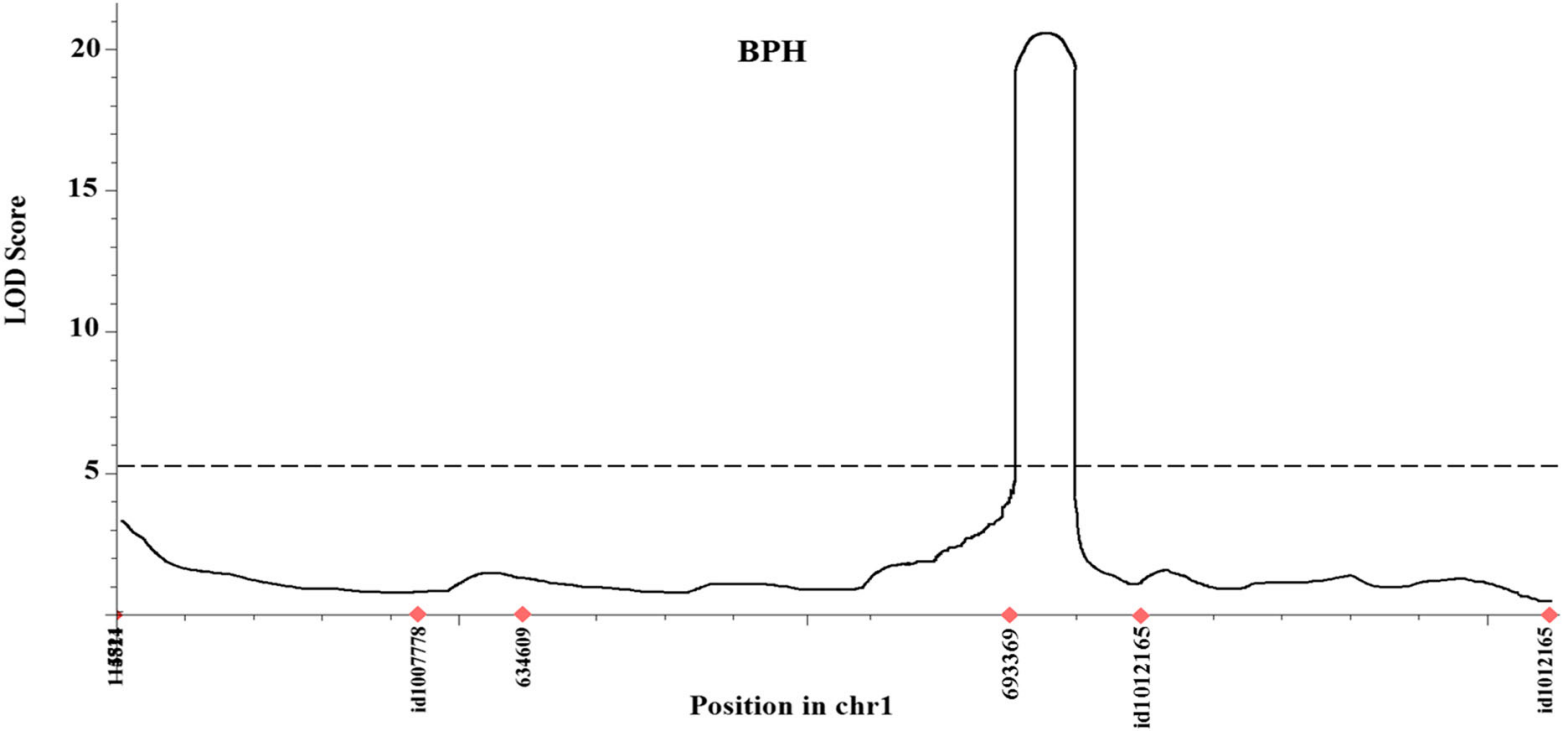
QTL map showing the detected BPH resistance locus, *BPH38*(t) derived from HHZ × Khazar

The precisely estimated BPH SES scores of 101 BC_1_F_5_ lines and genotypic data from high-quality SNP markers were used for the molecular mapping of the genomic regions conferring BPH resistance. Composite interval mapping with the 1000 permutation test was considered for the QTL analysis to make it more stringent. This analysis resulted in the identification of a putative locus showing as high as 35.91% PVE with LOD of 20.53. This locus was detected on chromosome 1 between SNP markers 693,369 and id 10,112,165, between the physical positions 20,706,894 bp and 21,203,101 bp of 496.2 kb in size in the reference genome sequences of Nipponbare (IRGSP 1.0) (Table 3 and Fig. 4).

**Table 3:** Details of the identified BPH resistance locus, *BPH38*(t) detected from the 101 BC_1_F_5_ mapping population derived from HHZ × Khazar

Detected locus	Chromosome	Position (cM)	Left marker	Right marker	LOD	PVE (%)	Add	Dom
*BPH38(t)*	1	112	693,369	id1012165	20.53	35.91	– 23.12	4.46

### Background analysis and phenotypic evaluation of the selected BC_1_F_6_ plants

**Fig. 5:**
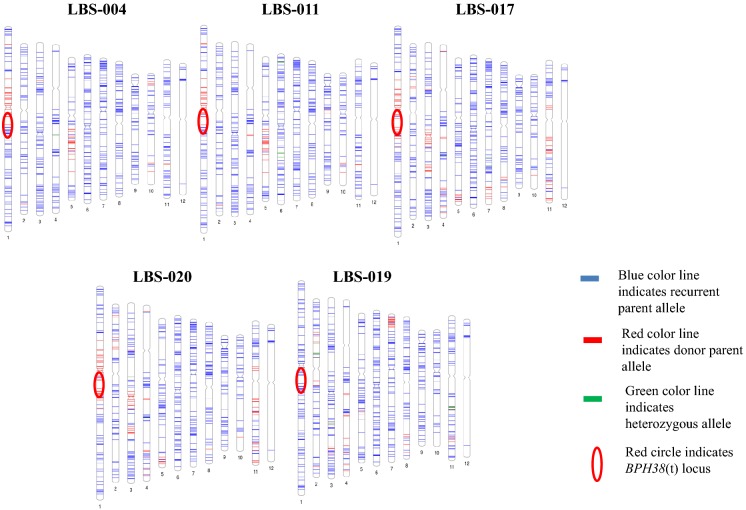
Graphical genotype map of selected BC_1_F_6_ plants. The map was constructed based on 702 high quality SNPs. LBS-004, LBS-011, LBS-017 and LBS-020 are the BC_1_F_6_ genotypes possessing *BPH38*(t) locus (positive ILs) whereas LBS-019 is the another BC_1_F_6_ doesn’t possess *BPH38*(t) locus (negative ILs). (Color figure online)

Six out of seven selected BC_1_F_6_ plants (from the 101 BC_1_F_6_ lines # LBS-004, LBS-009, LBS-011, LBS-013, LBS-017, and LBS-020) were carrying homozygous dominant allele of *BPH38*(t) locus (RR) while one BC_1_F_6_ plant (# LBS-019) was carrying homozygous recessive allele (rr). All the seven BC_1_F_6_ plants underwent background analysis using 702 SNP markers and were further evaluated to confirm their resistance reaction against BPH infestation. Background analysis was carried out to compute the percent recovery of the recurrent parent genome and size of the donor parent segment. The percent recovery of the recurrent parent genome ranged from 85.16 to 91.91% among the selected BC_1_F_6_ lines possessing *BPH38*(t) locus. The IL LBS-013 showed the highest percent recovery of the recurrent parent genome (91.91%), whereas LBS-017 showed the lowest percent recovery (85.16%). However, except for line LBS-019, all other ILs showed the introgressed donor segment of 496.2 kb in size, thus confirming the introgression of the donor segment (Fig. 5). Hence, as expected, all of the ILs except for LBS-019 showed an SES score of 3, indicating a high BPH resistance reaction and as the effect of the detected BPH resistance locus (Table 4).

**Table 4:** Background analyses and BPH resistance score of advanced ILs derived from HHZ × Khazar

S. No	Genotype	Total polymorphic SNP markers	Number of SNP allele			Percentage of recurrent parent genome recovered	*BPH35(t)* locus	BPH SES score
			HHZ	Khazar	Hetero			
1	LBS-004	702	642	56	2	91.85	Presence	3
2	LBS-011	702	637	58	6	91.29	Presence	3
3	LBS-017	701	595	102	4	85.16	Presence	3
4	LBS-020	702	605	95	2	86.32	Presence	3
5	LBS-009	702	634	54	8	91.66	Presence	3
6	LBS-013	702	639	53	7	91.91	Presence	3
7	LBS-019	702	638	47	13	92.33	Absence	7

Further, the 101 BC_1_F_6_ individuals were evaluated to assess their performance for key agro-morphological traits such as DFF, PH, TN, GY, and PY, along with donor and recurrent parents (Table 5). All the selected ILs exhibited taller plant type ranging from 99.11 cm to 115.20 cm compared with the recurrent parent HHZ (91.51 cm). A significant difference was observed for TN per plant ranged from 12.61 to 14.81, whereas the recurrent parent HHZ showed 12 TN per plant. For GY per plant and PY, two ILs (LBS-011and LBS-009) exhibited an advantage over HHZ. Three ILs (LBS-004, LBS-020, and LBS-013) were isophenic, and the remaining two ILs (LBS-017 and LBS-019) showed significantly lower GY. For DFF, IL LBS-019 (80 days) showed significantly lower DFF than HHZ (94 days), as shown in Table 5.

**Table 5:** Agronomic performance of selected ILs derived from HHZ × Khazar

S. No	Genotype	Plant height (cm)	Tiller number	Grain yield (g)	Plot yield (Kg)	Days to 50% heading
1	LBS-004	112.40	13.80	298	0.30	114
2	LBS-011	108.20	14.40	532	0.53	116
3	LBS-017	99.00	14.80	223	0.22	114
4	LBS-020	104.80	12.60	292	0.29	112
5	LBS-009	110.40	13.40	459	0.46	114
6	LBS-013	106.80	13.40	285	0.29	114
7	LBS-019	115.20	13.20	205	0.21	80
8	HHZ	91.50	12.00	290	0.29	94
9	IR50	96.33	15.20	387	0.39	109
	SD	5.78	1.39	28.41	0.15	2.29
	SEm	1.06	0.25	147.61	0.03	0.44
	Range	22.20	6.20	514.00	0.51	7.00

### Candidate gene analysis

In silico candidate gene analysis was performed by using online MSU and RiceXpro databases, and in these 71 candidate genes present within the vicinity of the flanking markers 693,369 and id 10,112,165 were retrieved. Most of them are expressed and retrotransposon proteins (Supplementary Table 3). Among all the candidate genes, *LOC_Os01g37260* is found near left SNP marker 693,369 (physical position 20,706,894 Mb) within a physical distance of 98.90 kb. This gene encodes OsFBX14, an F-box protein of FBXL class containing the LRR domain, which is proven to be involved in biotic stress resistance (Jin et al. 2004; Jain et al. 2007). However, research is ongoing to test these candidate genes for their relevance in the phenotypic expression of BPH resistance.

## Discussion

The increased density of BPH populations causes “hopper burn” symptom, which ultimately leads to complete death of rice plants (Prahalada et al. 2017). Although several strategies are available to manage BPH infestation, building BPH resistance in rice plants by identifying and introgressing BPH resistance genes is the most convenient and efficient strategy. To date, 37 BPH resistance genes have been identified from cultivated and wild species of *Oryza*:*BPH1*, *BPH2*, *BPH3*, *BPH4, BPH5, BPH6, BPH7, BPH8, BPH9, BPH10, BPH11*(t)*, BPH12*(t)*,BPH12, BPH13*(t), *BPH14*, *BPH15*, *BPH16*(t), *BPH17, BPH18, BPH19*(t)*, BPH20, BPH21, BPH22*(t)*, BPH23*(t)*,BPH24*(t)*, BPH25*(t)*, BPH26*(t), *BPH27*, *BPH28, BHP29,BPH30, BPH31, BPH32,BPH33*, *BPH3* and *BPH35, BPH36* and *BPH37* (Khush et al. 1985; Kabis and Khush 1988; Nemoto et al. 1989; Ishii et al. 1994; Murata et al. 1998; Hirabayashi et al. 1998; Renganayaki et al. 2002; Yanget al. 2002; Sharma et al. 2003; Yang et al. 2004; Hirabayashi et al. 2004; Sun et al. 2005; Chang-Chao et al. 2006; Chen et al. 2006; Jena et al. 2006; Sai Harini et al 2010; Jairin et al. 2007, 2010; Li et al. 2019; Ram et al. 2008; Rahman et al. 2009; Du et al. 2009; Qiu et al. 2010; Deen et al. 2010; Qiu et al. 2012; Myint et al. 2012; Huang et al. 2013; Wu et al. 2014; Wang et al. 2015; Wang et al. 2018; Ren et al. 2016; Prahalada et al. 2017; Kumar et al. 2018; Naik et al. 2018; Yang et al. 2019; Yuexiong et al. 2019). Among these, only eight genes (*BPH14, BPH17, BPH18, BPH26, BPH29, BPH9, BPH32, BPH31* and *BPH6*) were cloned and characterized (Du et al. 2009; Tamura et al. 2014; Liu et al. 2015; Wang et al. 2015; Ji et al. 2016; Ren et al. 2016; Zhao et al. 2016; Guo et al. 2018). However, most of the mapped resistance genes were only effective against a single BPH population/biotype (Horgan et al. 2015). Notably, several BPH resistance genes have been broken down by the evolution of new BPH biotypes, resulting in significant losses of rice production (Liu et al. 2015). Hence, it is imperative to identify new sources of BPH resistance and use them in a crop improvement program. In our study, we carried out high-resolution mapping of BPH resistance derived from Khazar and HHZ and mapped a new BPH resistance locus, *BPH38*(t), on the long arm of chromosome 1. This locus is located between SNP markers 693,369 and id 10,112,165, within the physical positions of 20.71 Mb and 21.23 Mb and having a size of 496.20 kb. This is the third BPH gene reported on chromosome 1 after *BPH33*(t), which was reported by Naik et al. (2018) within the physical region of 24.52 Mb and 25.51 Mb, and, recently, Yang et al. (2019) reported a novel QTL *BPH35* on chromosome 1 between RM302 and YM35 markers with in a physical distance of 32.98 Mb using IR64 as donor parent derived population. However, the location of the reported *BPH38*(t) locus in our study is novel as this is the first report on the detected locus from the donor. Early backcross generation BC_1_F_5_ plants derived from the novel Green Super Rice breeding strategy were used for the localization of genomic regions conferring BPH resistance. Analogous to our study, several researchers also used early backcross progenies for molecular mapping for locating the genomic regions conferring several beneficial traits. Tamura et al. (1999) detected the *Grh1* gene on chromosome 1 by using a backcross population and applying RFLP markers and linkage mapping. Similarly, one of the most used broad-sense bacterial blight (BB) resistance genes, *Xa27*(t), was also identified using an early backcross generation. *Xa27*(t) was transferred from tetraploid wild rice *Oryza minuta* into cultivated *O. sativa* and is being used in several BB resistance breeding programs (Gu et al. 2005).

The bioassay was conducted using the BPH colony collected from Laguna Province of the Philippines popularly called the Laguna colony. The bioassay revealed that the parent Khazar was the source for BPH resistance with a BPH SES score of 3 and the recurrent parent HHZ exhibited a susceptible reaction with a score of 9. The individuals of the mapping population exhibited a wide range of BPH SES scores ranging from 3 to 9. The frequency distribution of the SES scores of the mapping population showed a bimodal type of segregation, indicating Mendelian segregation of the BPH resistance. Furthermore, BPH resistance scores were tested for the chi-square goodness of fit, which also confirmed the monogenic inheritance of the BPH resistance factor (χ^2^_(0.52)_ = 1.62; Table 1). This result is also supported by the QTL mapping result, which showed high LOD and PVE for the identified *BPH38*(t) (Table 3; Fig. 4).

Several studies describe the mechanism of BPH resistance by cloning and characterizing several BPH resistance genes. These studies describe the BPH resistance influenced by candidate genes and their association with the chemical composition of the phloem sap, especially primary and secondary dietary metabolites. Salicylic acid (SA) is one of the metabolites reportedly involved in the BPH resistance mechanism (Du et al. 2009). The first cloned *BPH14* resistance gene and later cloned, *BPH26, BPH29, BPH9,* and *BPH18* were found to be involved in triggering the induction of SA through the signaling pathway and callous deposition in the phloem tissues coupled with trypsin secretion, which causes inhibition of sucking in the phloem and, hence, resistance (Du et al. 2009; Liu et al. 2015; Wang et al. 2015; Zhao et al. 2016; Ji et al. 2016; Prahalada et al. 2017). Similar to these researches, we have also explored the detected BPH resistance locus to identify the probable candidate gene involved in BPH resistance by applying recent genomics and bioinformatics tools. A genomic segment of *BPH38*(t) of 496.20 kb in size mapped in this study contained a minimum of 71 candidate genes. Most of the candidate genes within the locus were reported to be an encoding group of retrotransposon proteins followed by expressed proteins and other different kinds of proteins that were not associated with any biotic resistance (Supplementary Table 3). Interestingly, in our current study, we have also found a candidate gene *LOC_Os01g37260,* which is ~ 90.61 kb near the detected *BPH38*(t) resistance locus that encodes for F-box protein. F-box proteins constitute a large family in eukaryotes and are characterized by a conserved F-box motif (approximately 40 amino acids) at their N terminus that interacts with Skp1p-cullin-F-box (SCF) protein, which is a major class of plant E3 ligases (Jain et al. 2007). The name F-box was given because it was identified first in the N-terminal region of cycline F (Bai et al. 1994). The C terminus of F-box proteins generally contains one or several highly variable protein–protein interaction domains, including leucine-rich repeat (LRR) (Jin et al. 2004). Furthermore, there are 687 F-box proteins, and they have ten different domains, out of which FBLD and FBXL are the F-box protein subfamilies that possess the LRR domain in nine different categories (Jain et al. 2007). The candidate gene *LOC_Os01g37260* is one among those F-box proteins (https://rice.plantbiology.msu.edu/cgi-bin/ORF_infopage.cgi?orf=LOC_Os01g37260.1) that possesses the LRR domain at the interaction with the C terminus, which is reported to be involved in the salicylic acid signaling pathway to attain high BPH resistance which consequently acts as a defense against BPH attack (Yan et al. 2009; Ji et al. 2016). However, more observations and further research are needed to confirm the involvement of the candidate gene *LOC_Os01g37260* in the expression of BPH resistance.

The recurrent parent HHZ is a commercially released mega-variety, originating from Guangdong Province of China that is susceptible to BPH. The ILs were generated by introgressing the *BPH38*(t) locus and initially subjected to background analysis to select the lines with maximum recovery of the recurrent parent genome, and the same set of lines was used for the bioassay to study the effectiveness of the *BPH38*(t) locus. The ILs LBS-013 and LBS-004 showed 91.91% and 91.85% recurrent parent genome, respectively, and were found to possess the *BPH38*(t) locus. Notably, positive ILs possessing *BPH38*(t) exhibited a high level of BPH resistance compared with that of the background line HHZ. On the contrary, ILs without *BPH38*(t) showed a similar level of BPH reaction (susceptibility) as HHZ confirmed the efficacy of the *BPH38(*t) locus (Table 4; Fig. 5). These lines also showed superior yield and yield-associated traits, especially LBS-011 and LBS-009, which showed the highest yield of 5.85 and 5.05 t ha^−1^, respectively. On the other hand, the IL LBS-019 showed the highest recurrent parent genome recovery (92.33%) without exhibiting any resistance against BPH as it did not carry the *BPH38*(t) locus and was also not superior in agro-morphological characters in comparison to ILs carrying the *BPH38*(t) locus (Table 5). One backcross was made and was followed by six generations of selfing, which led to the higher recurrent parent genome recovery levels (85.16–91.91%) (Table 4; Hasan et al. 2015). Thus, our study not only detected a locus conferring BPH resistance but could also able to identify ILs that were ready to be used as breeding products. Hence, we strongly believe that the achievements of our study can help manage the BPH insect pests to a great extent.

## Electronic supplementary material

Below is the link to the electronic supplementary material.

Supplementary file1 (DOCX 22 kb)
