# Supplementary material for: Identification of a novel locus, BPH38(t), conferring resistance to brown planthopper (Nilaparvata lugens Stal.) using early backcross population in rice (Oryza sativa L.)
Source: Euphytica. 2019 Oct 10;215(11):185. doi: 10.1007/s10681-019-2506-2 (PMC6913135; doi:10.1007/s10681-019-2506-2)
Supplement: Supplementary file 1 — Supplementary file1 (DOCX 22 kb) [file 10681_2019_2506_MOESM1_ESM.docx]

**Supplementary Table 1** Scoring pattern based on seeding survival rate

| S. No | Seedling survival rate | Score | Remarks |
| --- | --- | --- | --- |
| 1 | 91–100% | 0-1 | Highly resistant |
| 2 | 76–90% | 2–3 | Resistant |
| 3 | 11–75% | 4-7 | Susceptible |
| 4 | 0–10% | 8-9 | Highly susceptible |

**Supplementary Table 2** Percent polymorphism of total SNPs utilized for the linkage analysis

| S. No | Chromosome number | Polymorphic SNPs | Total SNPs | Percent polymorphism |
| --- | --- | --- | --- | --- |
| 1 | 1 | 96 | 514 | 18.68 |
| 2 | 2 | 58 | 457 | 12.69 |
| 3 | 3 | 77 | 486 | 15.84 |
| 4 | 4 | 49 | 421 | 11.64 |
| 5 | 5 | 51 | 378 | 13.49 |
| 6 | 6 | 76 | 376 | 20.21 |
| 7 | 7 | 76 | 367 | 20.71 |
| 8 | 8 | 63 | 345 | 18.26 |
| 9 | 9 | 50 | 300 | 16.67 |
| 10 | 10 | 33 | 276 | 11.96 |
| 11 | 11 | 60 | 346 | 17.34 |
| 12 | 12 | 13 | 340 | 3.82 |

**Supplementary Table 3** Types of proteins encoded by candidate genes detected within the *BPH35*(t) locus

| S. No | Type of protein | Total in number |
| --- | --- | --- |
| 1 | ABIL2 | 1 |
| 2 | Agenet domain containing protein | 1 |
| 3 | asp/Glu racemase | 1 |
| 4 | Chaperone protein dnaJ 49 | 1 |
| 5 | Dynein light chain type 1 domain containing protein | 1 |
| 6 | Glutamate dehydrogenase protein | 1 |
| 7 | Glutathione S-transferase | 1 |
| 8 | Hypothetical protein | 1 |
| 9 | M16 domain containing zinc peptidase | 1 |
| 10 | Lucine Rich Repeat domain | 1 |
| 11 | OsFBX15 - F-box domain containing protein, | 1 |
| 12 | Oxido reductase/ transition metal ion binding protein | 1 |
| 13 | Peptide deformylase | 1 |
| 14 | Peptide transporter PTR2 | 1 |
| 15 | PPR repeat domain containing protein | 1 |
| 16 | Protein phosphatase 2C | 1 |
| 17 | Ras-related protein | 1 |
| 18 | RWD domain containing protein | 1 |
| 19 | RWP-RK domain-containing protein | 1 |
| 20 | RWP-RK domain-containing protein | 1 |
| 21 | Sodium/calcium exchanger protein | 1 |
| 22 | SWIM zinc finger family protein | 1 |
| 23 | Thioredoxin, putative | 1 |
| 24 | WD domain, G-beta repeat domain containing protein | 1 |
| 25 | Zinc finger family protein | 1 |
| 26 | Esterase | 2 |
| 27 | Transposon protein, putative | 5 |
| 28 | Expressed protein | 18 |
| 29 | Retrotransposon protein | 21 |
